# Neck Dissection for Thoracic Esophageal Squamous Cell Carcinoma

**DOI:** 10.1155/2012/750456

**Published:** 2012-02-27

**Authors:** Satoshi Yajima, Yoko Oshima, Hideaki Shimada

**Affiliations:** Department of Surgery, Toho University Omori Medical Center, 6-11-1 Omori-Nishi, Ota-Ku, Tokyo 143-8541, Japan

## Abstract

Subtotal esophagectomy with extended lymph node dissection is a standard procedure for thoracic esophageal squamous cell carcinoma. Three-field lymphadenectomy, including neck dissection, is a standard type of lymph node dissection for complete clearance of tumor cells. Based on various series of analyses for lymph node metastases, the appropriate indication for neck dissection has been clarified. Herein, we describe the established techniques of neck dissection and review recent topics of three-field lymph node dissection for thoracic esophageal squamous cell carcinoma.

## 1. Introduction

Neck dissection was introduced in the early 1980s based on analyses of the spreading pattern of lymph node metastases from thoracic esophageal squamous cell carcinoma (SCC) [1, 2]. Extended lymph node dissection including neck, mediastinal, and abdominal lymph nodes has been called three-field lymph node dissection (3FLD) [3]. The neck dissection includes cervical paraesophageal nodes, deep cervical nodes, and supraclavicular nodes, and it was subsequently adopted as the standard surgical procedure for thoracic esophageal SCC at high-volume centers in Japan in the late 1980s [4, 5]. A similar surgical procedure was also introduced in Western nations in the late 1990s [6, 7].

## 2. Indications for Neck Dissection

Despite recent advances in preoperative staging, including computed tomography, positron emission tomography, and ultrasonography, the pathological findings after 3FLD indicate that the overall accuracy, sensitivity, and specificity of clinical staging remain too low. More than 75% of cervical metastases are not detected before surgery. Therefore, the indication for neck dissection should be determined by tumor location, tumor depth, and/or intraoperative nodal assessment of recurrent nerve nodes. A summary of the prevalence of positive cases of cervical lymph node metastases and overall 5-year survival after 3FLD is shown in Table 1.

The prevalence of positive cervical lymph nodes associated with tumors in the upper part of the thoracic esophagus was significantly higher than that derived from tumors in the lower part of the esophagus (upper, 48%; middle, 28%; lower, 13%) [4]. Among the patients with lower third esophageal tumors, none of those with T1b tumors had cervical metastasis, whereas 16% of those with T2-T4 tumors did. Moreover, 29% of patients with tumors in the upper third of the esophagus had initial lymph node metastases into the cervical field according to the data of patients with solitary lymph node metastases. This prevalence was significantly higher than that of patients with tumors in the middle or lower third section of the esophagus (13% and 0%, resp.). Recurrent nerve nodes may be sentinel nodes that predict cervical lymph node metastases in patients with tumors in the middle or lower third of the esophagus [8]. Although neck dissection became a standard procedure for upper third tumors, this procedure is not essential for lower third tumors. Application for middle third tumors is still controversial. Overall survival was significantly better in the 3FLD group than in the two-field lymph node dissection (2FLD) group (without neck dissection) if the patients had recurrent nerve lymph node metastases, but there was no significant difference between the two groups if the patients did not have recurrent nerve lymph node metastases [8]. Neck dissection, therefore, may improve survival only for patients with recurrent nerve node metastases.

## 3. Surgical Procedure of Neck Dissection

**Figure 1:**
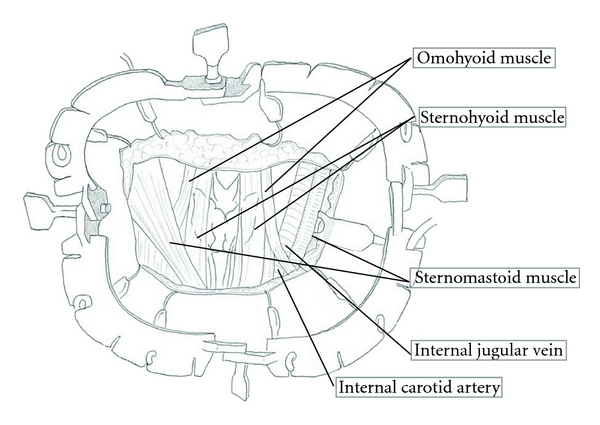
Lone Star Retractor System to make operative field.

**Figure 2:**
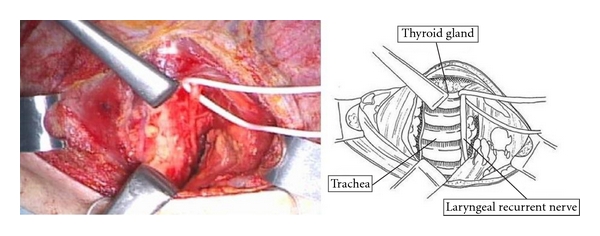
Dissection of left cervical paraesophageal lymph nodes. Left laryngeal recurrent nerve was taped.

**Figure 3:**
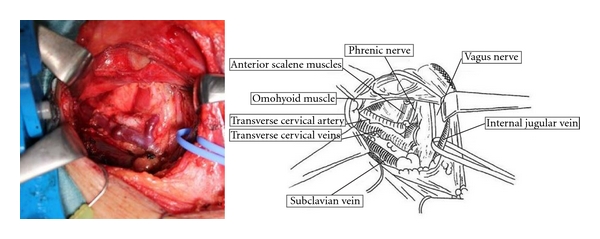
Dissection of the right side supraclavicular lymph nodes.

A summary of neck dissection for thoracic esophageal squamous cell carcinoma has been described [4, 5]. Patients were placed in the supine position so that a U-shaped neck incision could be made. Through the cervical incision, the Lone Star Retractor System (Cooper Surgical, Inc.) was used to make the operative field (Figure 1). We performed mediastinal lymph node dissection through a right transthoracic approach before neck dissection, so the remainder of the recurrent nerve nodes was located posterior and lateral to the carotid sheath. Thus, the cervical lymph nodes included a continuous, anatomically inseparable chain of nodes that extended from the superior mediastinum to the lower neck (Figure 2). The sternomastoid and strap muscles were preserved, and the cervical nodes (internal jugular nodes below the level of the cricoid cartilage, supraclavicular nodes, and cervical paraesophageal nodes) were removed bilaterally (Figure 3). The paraesophageal nodes (including the recurrent nerve nodes in the cervicothoracic junction) were classified as cervical or upper mediastinal nodes according to their position relative to the bifurcation between the right common carotid and right subclavian artery.

## 4. Survival after 3FLD

The 5-year survival in consecutive studies after the introduction of neck dissection was 34–52%, with surgical mortality values of 1.2–5%. The overall 5-year survival was 57–88% for node-negative patients and 25–37% for patients with nodal metastases [3–5]. Although lymph node metastases into the cervical field are classified as distant metastases, 13–32% of patients survived >5 years after clearance of positive cervical nodes [3–5]. Similar survival values were obtained in recent Western studies reported by Altorki et al. [6] and Lerut et al. [7]. More recent series also showed good survival curves in patients with positive neck lymph nodes, which were equivalent to those of patients with positive mediastinal nodes [9].

## 5. Survival of Patients with Positive Neck Lymph Nodes

Among cervical node-positive patients, those with tumors in the upper esophagus showed significantly better survival than those with tumors in the lower esophagus [4]. Hence, cervical nodes may be regional nodes that should be routinely dissected (at least in patients with tumors in the upper part of the esophagus). The number of patients with abdominal node metastases from upper esophageal tumors was small, so a significant association between abdominal node-positive patients and tumor location was not observed. The real impact of neck dissection on overall survival should be evaluated by randomized controlled clinical trials. Two randomized trials were performed in Japan by Kato et al. [10] and Nishihira et al. [11], who compared 2FLD and 3FLD from the late 1980s to early 1990s. Both studies showed that extended lymphadenectomy can prevent recurrence and prolong survival after resection of thoracic esophageal carcinoma, with an advantage of 3FLD over 2FLD. The overall 5-year survival for 3FLD and 2FLD patients was 49% and 33% (*P* < 0.01) [10] and 66% and 48% (*P* = 0.19) [11]. Extended lymphadenectomy increased the R0 resection rate at lymph node levels, which seemed to reduce the incidence of locoregional recurrence, and the available data suggested a potential survival benefit for 3FLD patients. It appears that positive cervical lymph nodes in patients with tumors in the upper third part of the esophagus should no longer be considered as distant metastases but rather as regional metastases.

## 6. Difficulties in Performing Randomized Trials

Upper mediastinal lymph node dissection and recurrent nerve node dissection are essential. However, the optimal indication for neck dissection for middle or lower third thoracic esophageal carcinoma remains controversial. There remains a need for randomized studies to compare 3FLD with 2FLD for middle or lower third esophageal carcinoma without clinically detectable cervical node metastases. Because of the difficulties involved in recruiting a sufficient number of patients and the technical difficulties associated with neck dissection, such randomized trials have not been performed in Western countries. Conducting prospective randomized trials to compare the survival curves for 2FLD and 3FLD groups will be difficult because most surgeons in high-volume centers in Japan prefer neck dissection for thoracic esophageal SCC.

## Figures and Tables

**Table 1:** Positive rates of cervical lymph node metastases and overall five-year survival rates after three-field lymph node dissection.

	Positive rates of cervical lymph node metastases according to tumor location				Overall 5-year survival rates according to pN status			
Authors	Upper (%)	Middle (%)	Lower (%)	Total (%)	pN0 (%)	pN1 (%)	pN1 in cervical field (%)	Total (%)
Isono et al. [3]	42	38	19	33	NA	NA	NA	34
Altorki et al. [6]	13	59	33	36	88	33	25	51
Lerut et al. [7]	44.4	26.2	23	23.6	80.2	24.5	12.8	41.9
Shiozaki et al. [8]	23.5	34.5	10.5	27.5	NA	NA	NA	48.5
Shimada et al. [4]	48	30	18	26	68	37	32	52
Tachimori et al. [9]	21.2	25.5	5.6	17	85	30~50	30	NA
